# What is the impact of intraperitoneal surfactant administration against postoperative intraabdominal adhesion formation? an experimental study

**DOI:** 10.55730/1300-0144.5752

**Published:** 2023-11-25

**Authors:** Şehmus PALA, Tuncay KULOĞLU, Remzi ATILGAN, Zehra Sema ÖZKAN, Serhat HANÇER

**Affiliations:** 1 Department of Obstetrics and Gynecology, Fırat University School of Medicine, Elazığ, Turkiye; 2 Department of Histology and Embryology, Fırat University School of Medicine, Elazığ, Turkiye; 3 Department of Obstetrics and Gynecology, Kırıkkale University School of Medicine, Kırıkkale, Turkiye

**Keywords:** Postoperative adhesion, uterine horn, rat, surfactant, experimental model

## Abstract

**Background/Aim:**

Surfactant is a surface-active substance that, in addition to its detergent effect, also has effects that reduce inflammation and fibrosis. Because of these effects, it was aimed herein to investigate the effect of intraperitoneal surfactant application on preventing postoperative peritoneal adhesion formation in a uterine horn adhesion model.

**Materials and methods:**

Twenty-one Wistar albino rats were randomly divided into 3 groups (G1–G3), as follows: G1 (n = 7): control group. The abdomen was opened and then closed; G2 (n = 7): adhesion group. The abdomen was opened. Then, a 2-cm linear incision was made over the right uterine horn, 2 mL of isotonic saline was administered intraperitoneally, and the abdomen was closed; and G3 (n = 7): treatment group. The abdomen was opened, a 2-cm linear incision was made over the right uterine horn, 2 mL (70 mg/kg) of surfactant was administered intraperitoneally, and the abdomen was closed. After 15 days, the rats were euthanized, the abdomens were reopened, and adhesion scoring was performed. After the right uterine horns were removed and fixed with 10% formalin, appropriate sections were taken from the traumatized tissue, stained with Masson’s trichrome, and fibrosis and inflammation scoring were performed.

**Results:**

The adhesion area and intensity were significantly higher in G2 than in G1 and G3 (p = 0.001) and were similar in G1 and G3 (p = 0.165). While fibrosis and inflammation were significantly higher in G2 than in G1 and G3 (p = 0.001), there was no difference between G1 and G3 (p = 0.5).

**Conclusion:**

Intraperitoneal surfactant administration at a dose of 70 mg/kg was found to be effective in preventing intraabdominal adhesion formation in a rat uterine horn model.

## 1. Introduction

Postoperative peritoneal adhesion (PPA) after abdominal surgery is a frequent complication that occurs between abdominal organs or tissues. PPA can cause complications that require urgent intervention, such as ileus and intestinal necrosis, as well as chronic pelvic pain and infertility in women [1]. It has been shown that, even with minimally invasive surgical techniques, the formation of PPA cannot be prevented [2].

It is a fact that unlimited physiological peritoneal healing due to increased vascular permeability leads to PPA. Although many medical agents and surgical techniques have been tried, there is currently no accepted standard management to prevent PPA formation. Due to ethical concerns, experimental animal models in which rats are generally preferred instead of humans are used in studies on PPA [3–6].

Within a few minutes after peritoneal injury, platelet adhesion and aggregation occur on the lesion with the activation of acute inflammation and coagulation. This results in an increase in blood flow and a fibrin mesh is formed as a result of vasodilation, increased vascular permeability, and migration of neutrophils and macrophages onto the lesion. Within a few hours, macrophages accumulate in the lesion and fibrinolytic activity begins [7]. Fibroblast and mesothelial cells activated by platelet-derived growth factors clear debris. Mesothelial cell growth begins to be detected after 24 h, fibroblast proliferation occurs on the 3rd day, and angiogenesis occurs on the 5th day [8–11]. Fibrinolysis and inflammation play important roles in adhesion development. A blockade of thrombin-activated fibrinolysis inhibitor reduces adhesion formation [12].

It has been reported that cleaning wounds and removing debris from the wound area may contribute to wound repair as well as removing microorganisms. This should be a strategic step in wound management [13]. Wound cleaners and dressings containing surfactants contribute positively to the wound healing process through autolytic debridement [14]. Since surfactants reduce the surface tension between liquid and liquid or liquid and solids, they are used as detergents, wetting agents, emulsifiers, and foaming agents [15]. Surfactant administration has been shown to reduce inflammation and fibrosis scores. It has been suggested that this antiadhesive effect reveals the effect of surfactant by reducing tissue inflammation and fibrosis due to its antiinflammatory effect and antifriction properties [16].

In this experimental uterine horn adhesion model study, it was aimed to investigate the potential of intraperitoneal surfactant application on the prevention of PPA formation.

## 2. Materials and methods

**Figure 1:**
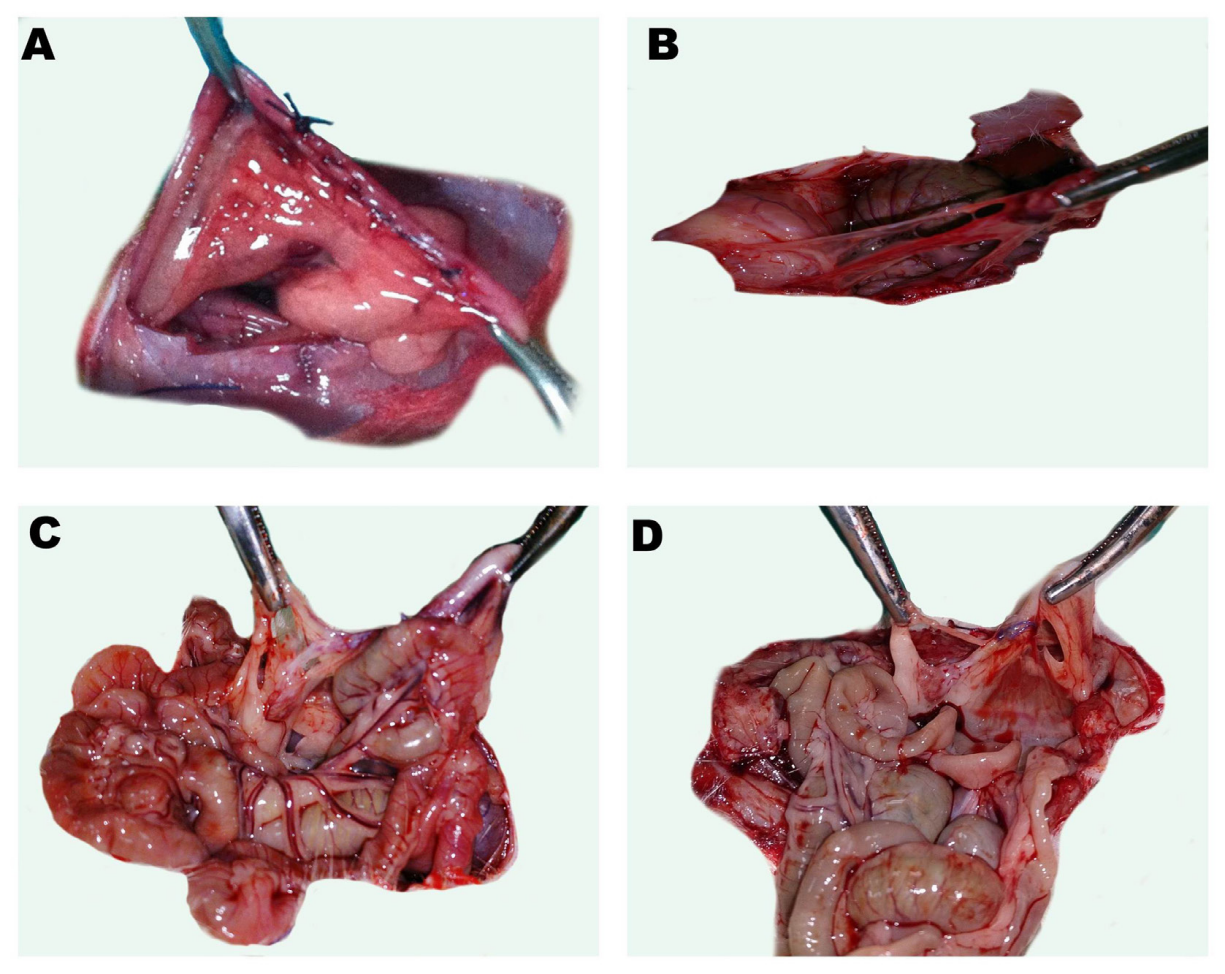
Intraabdominal macroscopic images from all 3 groups. A) Image of the incision made to form adhesion to the uterine horn. B) Image from G1. Film adhesions are observed between the abdominal wall and the intraabdominal organs. C) Image from G2. Wide-surfaced, dense adhesions are observed in the uterine horn, including the surrounding tissue and the intestinal loop. D) Image from G3. Film adhesion is observed between the uterine horn and the surrounding tissue.

This experimental single-blind, randomized controlled study was carried out in Fırat University Experimental Animals Laboratory between 03/10/2020 and 03/30/2020. In this study, 21 female Wistar Albino rats, aged 14 weeks with regular cycles and weighing 200 ± 40 g, were used. They were kept in room with a 12 (08:00–20:00 h)/12 light/dark photoperiod and a constant temperature of 21–23 °C and fed standard pellet food and water ad libitum. Thus, the rats were subjected to a 1-week adaptation period. Permission for this study was obtained from the local Animal Study Ethical Committee of Fırat University, Faculty of Medicine (Approval Date: 03/06/2013; Number: 2013/02; Decision Number: 26). Interventions applied to the rats were carried out in accordance with the rules of the local ethics committee. Anesthesia was achieved via the intramuscular administration of 60 mg/kg ketamine (Ketalar, Eczacıbaşı Warner-Lambert, İstanbul, Türkiye) and 7 mg/kg xylazine (Rompun, Bayer, İstanbul, Türkiye) into the left hind foot muscle. The rats were placed on the operating table in the supine position, the surgical area was washed with 10% povidone iodine solution to provide antisepsis, and the abdomens were opened with a midline incision. The rats were divided into 3 groups (G1–G3), as follows:

G1 (n = 7): control group. The abdomen was opened and then closed (sham group).
G2 (n = 7): adhesion group. The abdomen was opened. Then, a 2-cm linear incision was made over the right uterine horn, 2 mL of isotonic saline (0.9% NaCl, Eczacıbaşi, İstanbul, Türkiye) was administered intraperitoneally, and the abdomen was closed with 4-0 polyglactin sutures.
G3 (n = 7): treatment group. The abdomen was opened, a 2-cm linear incision was made over the right uterine horn, 2 mL (70 mg/kg) of surfactant (beractant (active ingredient); Survanta, Abbvie Inc., North Chicago, IL, USA), was administered intraperitoneally, and the abdomen was closed with 4-0 polyglactin sutures (Figure 1A).

The intraperitoneal administration of 70 mg/kg of surfactant was diluted with 0.9% NaCl to 2 mL (25 mg/mL) [Survanta was in the form of a sterile suspension presented in single-use glass vials containing 8 mL (200 mg phospholipid). Beractant is the active ingredient in Survanta; phospholipids (25 mg/mL), free fatty acids (1.4–3.5 mg/mL), triglycerides (0.5–1.75 mg/mL), and protein (0.1–1.0 mg/mL)].

No antibiotics or analgesics were used during the experimental period.

The abdominal layers were closed with 3-0 silk. The abdomens of the rats were reopened with a vertical incision 15 days later. Abdominal cavity was observed. Adhesion scoring was done blindly by one of the authors using the Linsky scale [17] (Table 1). At the end of the experiment, all of the rats were euthanized.

### 2.1. Histological examination

The uterine horn area, including the adhesion area, was quickly removed, and fixed with 10% formaldehyde and then embedded in paraffin blocks. Then, 5–6-μm-thick sections were taken from the paraffin blocks and hematoxylin and eosin (H&E) and Masson’s trichrome staining were applied. Histopathological postoperative adhesion scoring was performed according to the scoring system defined by Hooker et al. [18] (Table 2).

### 2.2. Statistical analysis

When a power analysis (G power, Germany) was performed at 80% power and 0.05 significance level for the variable with the largest standard deviation among the variables to be used in the study, it was calculated that there should be at least 5 subjects in each group and 7 optimally. IBM SPSS Statistics for Windows 21.0 (IBM Corp., Armonk, NY, USA) was used for the statistical evaluation. Determining the normality of the data was performed with the Shapiro–Wilk test and the descriptive statistics were expressed as the median (minimum and maximum). Comparison of the continuous variables among the 3 groups was done with the Kruskal–Wallis analysis of variance with post hoc comparisons with the Mann–Whitney U test. p < 0.05 was accepted as statistically significant.

## 3. Results

The experiment performed for adhesion formation was successful in all of the rats. The comparison of the adhesion area among the groups revealed that the surfactant significantly decreased the adhesion area when compared to G2 group (Table 3). When G1 (Figure 1B) and G2 (Figure 1C) were compared, the adhesion area was significantly larger in G2 (p = 0.001). When G1 (Figure 1B) and G3 (Figure 1D) were compared, the adhesion area was similar in both groups (p = 0.165). When G2 (Figure 1C) and G3 (Figure 1D) were compared, the adhesion area in G3 was significantly smaller than in G2 (p = 0.001).

All of the scores obtained from staining of the adhesion tissue with H&E staining and Masson’s trichrome staining under light microscopy are shown in Table 4.

**Figure 2:**
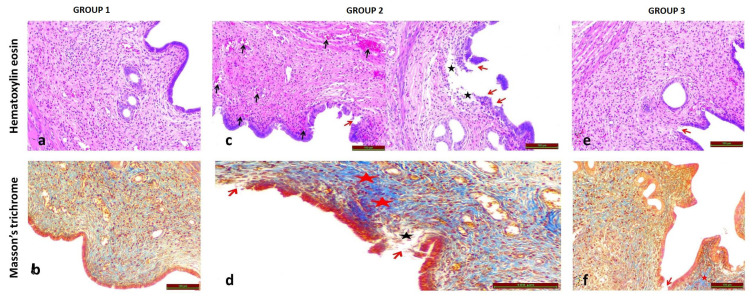
Histopathological images from all 3 groups (H&E and Masson’s trichrome staining): a–b) G1, c–d) G2, and e–f) G3. When G1 and G2 are compared, a significant increase in congestion (black arrow), edema (black star), epithelial separation and deterioration (red arrow) and fibrosis (red star) are observed in G2 compared to G1. Compared with G2, a significant reduction in the aforementioned histopathological findings can be seen in G3.

Histological images of G1 are shown in Figures 2a (H&E) and 2b (MT).

Histological images of G2 are shown in Figures 2c (H&E) and 2d (MT).

Histological images of G3 are shown in Figures 2e (H&E) and 2f (MT).

Congestion: When comparing G1 (Figures 2a and 2b) with G2 (Figures 2c and 2d), the congestion score was significantly higher in G2 (p = 0.001). When comparing G1 (Figures 2a and 2b) with G3 (Figures 2e and 2f), there was no statistically significant difference between congestion scores (p = 0.6). When G2 and G3 were compared, the congestion score was significantly higher in G2 (p = 0.006).

Edema: When G1 and G2 were compared, the edema score was significantly higher in G2 (p = 0.001). There was no statistically significant difference between the edema scores when G1 and G3 were compared (p = 0.7). When G2 and G3 were compared, the edema score was significantly higher in G2 (p = 0.003).

Epithelial degeneration: When G1 and G2 were compared, the epithelial degeneration score was significantly higher in G2 (p = 0.001). There was no statistically significant difference between the epithelial degeneration scores when G1 and G3 were compared (p = 0.9). When G2 and G3 were compared, the epithelial degeneration score was significantly higher in G2 (p = 0.004).

Fibrosis: When G1 and G2 were compared, the fibrosis score was significantly higher in G2 (p = 0.001). There was no statistically significant difference between the fibrosis scores when G1 and G3 were compared (p = 0.5). When G2 and G3 were compared, the fibrosis score was significantly higher in G2 (p = 0.006).

Inflammation: When G1 and G2 were compared, the inflammation score was significantly higher in G2 (p = 0.001). There was no statistically significant difference between the inflammation scores when G1 and G3 were compared (p = 0.5). When G2 and G3 were compared, the inflammation score was significantly higher in G2 (p = 0.007).

## 4. Discussion

In this study it was observed that intraperitoneal surfactant application significantly reduced the area and severity of postoperative intraabdominal adhesion in the adhesion model created by trauma to the uterine horn. This effect can be attributed to the detergent effect of the surfactant as well as the reduction of inflammation and fibrosis.

It has been shown that the addition of phospholipids that facilitate intestinal gliding after surgery can reduce the formation of PPA by 30% [19–21]. If viscous fluids are administered, particularly before mechanical manipulation, adhesion formation can be reduced, possibly as a result of a reduction in mechanical trauma [22,23]. Surfactant was also applied before applying the uterine horn adhesion procedure in the current experiment. A significant increase was found in the fibrosis and inflammatory cells in the G2 compared to the G1.

Although surfactants such as poloxamers have been shown to aid in wound healing in clinical and in vitro studies [24,25], their mode of action and interactions with antimicrobials and antibiofilm agents are not fully known. Surfactants aid in wound cleansing. In addition, they suppress protein aggregation and denaturation, seal tissue or cell membranes, and play a role in repair. Surfactants can also stabilize antimicrobials and exert antimicrobial activity [14].

Phospholipids, which are surfactants and excellent lubricants, temporarily cover serosal defects [26]. These endogenous phospholipids covering the peritoneal surface are hydrolyzed by phospholipases during peritoneal healing [27]. Exogenously applied phospholipids can replace the hydrolyzed endogenous phospholipid layer that covers the mesothelium [28]. The surfactant used herein contained 25 mg/mL of phospholipid. Therefore, the surfactant that was applied on the traumatized area before uterine horn trauma can have the same effects as above.

Intraperitoneal administration of a single dose of phosphatidylcholine (PC) at a dose of 70 mg/kg was shown to reduce adhesion formation in the current study. In contrast, intravenous administration of PC, even at very high doses such as 70 or 180 mg/kg, has been shown to be ineffective in preventing adhesion formation [29]. Interestingly, the administration of PC at doses greater than 140 mg/kg has been reported to result in increased anastomotic leak rates and peritonitis-related deaths [30]. It was concluded that the lack of antiadhesive effect at high doses is due to the overactivation of the antiadhesive capacity by disrupting the normal tissue adhesion at the wound site for adequate anastomosis healing [31]. In a rabbit trial involving a complex abdominal surgical procedure, a single intraperitoneal dose of 120 mg/kg of phospholipids was much higher than the dose considered sufficient to prevent adhesions [32]. To determine the ideal phospholipid dosage, a dose-dependent study compared the efficacy of a single intraabdominal administration of phospholipids at doses of 30 and 70 mg/kg. As a result of the study, a dosage of 70 mg/kg was shown to be more effective than 30 mg/kg. However, the healing process of surgical lesions up to 10 days postoperatively was not affected by the dosage regimen [33]. In a metaanalysis of 24 experimental studies, the use of phospholipids was shown to be effective in preventing adhesions. A single intraperitoneal dose of approximately 75 mg/kg of PC with a 30-min exposure time was established as the standard administration dose for efficacy, both in surgery alone and in combination with peritonitis [34]. The absence of any impairment in wound healing indicates that this agent is safe. It has been shown that intraabdominally administered phospholipids, in addition to inhibiting bacterial adhesion and proliferation, can eliminate peritoneal carcinoma by inhibiting the intraperitoneal adhesion of tumor cells [21]. It was reported that this effect can only be achieved with a phospholipid dose equal to 150 mg/kg (21). These experimental data support the intraperitoneal administration of phospholipids to prevent adhesion formation following intraabdominal surgical trauma without significant overdose-related adverse effects. In addition, it was reported that these substances can possibly reduce posttraumatic inflammation and inhibit intraperitoneal tumor cell adhesion [35]. In the current study, it was observed that the dose of 70 mg/kg of surfactant administered intraperitoneally significantly reduced the PPA area and severity. When the rats were opened for the second time on the 15th day, it was observed that wound healing had taken place. Therefore, it was not possible to have information about the wound status on the 10th day.

Beractant, a pulmonary surfactant, is the active ingredient of surfactant and is used to treat respiratory distress syndrome in premature infants. It consists of surfactant phospholipids, especially PC, which is the main component of the surfactant coating the peritoneal mesothelium [36]. PC is an excellent lubricant and forms a temporary membrane by covering the entire traumatized peritoneum, thus exerting its antiadhesive effect [20,29]. Chen et al. [37] demonstrated the absorption of phospholipids by the mesothelium and then the formation of a thin membrane-like layer over the entire peritoneum. They stated that the PC-rich layer acts as a gliding fluid barrier and reduces adhesion formation during the healing process [20,21,37]. Arslan et al. [16] showed that beractant application significantly reduces adhesion formation by 70% with an adhesion score of 0. Just before the surgical intervention, the uterine horn incision was filled with surfactant and then the surgical procedure was performed. Afterwards, 1 mL of surfactant mixture was left in the abdomen to cover the entire abdominal peritoneum and the abdomen was closed. Thus, the formation of a phospholipid film layer on the peritoneal surface was ensured. Hence, it was shown that this application method and the dosage that was applied is an effective method for preventing intraperitoneal adhesion in a rat model.

Minimal traumatic manipulation is the most important step to prevent PPA. In addition to this strategy, various methods such as frequent irrigation, placing mechanical barriers such as the film, solution, and gel type on the damaged tissue surface, and applying chemical barriers such as statin, nonsteroidal antiinflammatory agents, and heparin have been used. In addition, fibrinolytic agents, thrombin-activated fibrinolysis inhibitors, and a combination of mechanical and chemical barriers have also been used. However, it has been reported that none of the antiadhesive agents used have perfect antiadhesive activity [38].

In a rat peritoneal adhesion model, polysaccharide films were shown to prevent PPA formation as successfully as Seprafilm [39]. In another study, the effectiveness of a new nanoengineered hydrogel in preventing PPA formation was investigated by combining suitable materials that can be injected and sprayed and have unique mechanical and biological properties that trigger biological responses to prevent PPA [40]. In another metaanalysis, the feasibility and superiority of using hydrogel to prevent PPA was confirmed [41]. In addition to antiadhesive agents produced with this nanotechnology, a metaanalysis showed that methylene blue has a beneficial effect on intraperitoneal adhesion after laparotomy and that adhesions decrease with an increasing dose [38]. As mentioned above, many experimental and clinical studies have been conducted to prevent PPA formation. In this context, it was shown in the experimental study herein that surfactant is effective in preventing PPA formation.

There were some limitations of this study. Beractant metabolism in rats is not fully known and the pathophysiological aspect of tissue healing may differ from that of humans. However, the high surface volume of the human abdomen may also increase the beractant dose requirement. Herein, no side effects related to beractant administration (70 mg/kg) were observed. However, the animal study results could not be projected directly to humans. Prospective clinical studies should be conducted to overcome these concerns. The strength of this study is the fact that animal studies are indispensable for adhesion experiments. The fact that it was shown that surfactant, in addition to its detergent effect, has the potential to prevent inflammation and fibrosis, which play a role in adhesion formation, shows that surfactant can be used as an antiadhesion agent. The presence of a detergent effect, and antiinflammatory and antifibrotic properties in a single agent will also be an advantage of surfactant use. A more effective antiadhesive effect can be achieved by adding surfactant to antiadhesive barrier agents.

In conclusion, intraperitoneal administration of 2 mL of surfactant, at a dose of 70 mg/kg, is effective in preventing PPA formation in rats. Adhesion barriers containing surfactant can be an effective method in preventing PPA formation. New studies are needed to discuss and project these results to humas.

## Figures and Tables

**Table 1:** Linsky scoring according to the adhesion area and severity.

Adhesion area	Score	Adhesion severity	Score
No adhesion	0	No resistance to separation	0
25% of the traumatized area	1	Moderate strength required	0.5
25%–50% of the traumatized area	2	Sharp dissection required	1
50%–100% of the traumatized area	3		

**Table 2:** Presence and extent of the congestion, edema, epithelial degeneration, and fibrosis, and the inflammation scores.

Congestion, edema, and epithelial degeneration	Fibrosis	Inflammation	Score
No	No	No	0
Mild	Mild	Presence of giant cells, occasional lymphocytes, and plasma cells	1
Moderate	Moderate	Presence of giant cells, plasma cells, eosinophils, and neutrophils	2
Severe	Severe	Presence of many inflammatory cells and microabscesses	3

**Table 3:** Comparison of the adhesion area and severity scores among the 3 groups.

Groups	Adhesion area	Adhesion severity
G1	0.00 (0.00–1.00)	0.00 (0.00–0.50)
G2	3.00 (1.00–3.00)^a^	1.00 (0.50–1.00)^a^
G3	1.00 (0.00–2.00)^b^	0.00 (0.00–0.50)^b^
p-value	0.001	0.001

Values are presented as the median (minimum–maximum). G1: sham group, G2: adhesion group, G3: surfactant group.

a G1 vs. G2, p = 0.001;

b G2 vs. G3, p = 0.001.

**Table 4:** Histopathological comparison of the adhesion area in terms of congestion, edema, epithelial degeneration, fibrosis, and inflammation.

Parameters	G1	G2	G3	p-value
Congestion	0.00 (0.00–1.00)	2.00 (2.00–3.00)^a^	0.00 (0.00–2.00)^b^	<0.001
Edema	0.00 (0.00–1.00)	3.00 (1.00–3.00)^a^	0.00 (0.00–1.00)^b^	<0.001
Epithelial degeneration	0.00 (0.00–1.00)	2.00 (2.00–3.00)^a^	0.00 (0.00–1.00)^b^	<0.001
Fibrosis	0.00 (0.00–1.00)	2.00 (2.00–3.00)^a^	0.00 (0.00–1.00)^b^	0.001
Inflammation	0.00 (0.00–1.00)	2.00 (2.00–3.00)^a^	0.00 (0.00–1.00)^b^	0.001

Values are presented as the median (minimum–maximum). Fibrosis: ^a^ G1 vs. G2, p = 0.001; ^b^ G2 vs. G3, p = 0.006. Inflammation: ^a^ G1 vs. G2, p = 0.001; ^b^ G2 vs. G3, p = 0.007. Congestion: ^a^ G1 vs. G2, p = 0.001; ^b^ G2 vs. G3, p = 0.006. Edema: ^a^ G1 vs. G2, p = 0.001; ^b^ G2 vs. G3, p = 0.003. Epithelial degeneration: ^a^ G1 vs. G2, p 0.001; ^b^= G2 vs. G3, p = 0.004. Kruskal–Wallis test.
